# ‘Pre-endoscopy point of care test (Simtomax- IgA/IgG-Deamidated Gliadin Peptide) for coeliac disease in iron deficiency anaemia: diagnostic accuracy and a cost saving economic model’

**DOI:** 10.1186/s12876-016-0521-5

**Published:** 2016-09-15

**Authors:** Michelle Shui Yee Lau, Peter Mooney, William White, Victoria Appleby, Sulleman Moreea, Ismail Haythem, Joshua Elias, Kiran Bundhoo, Gareth Corbett, Liam Wong, Her Hsin Tsai, Simon Cross, John Hebden, Sami Hoque, David Sanders

**Affiliations:** 1 Academic Department of Gastroenterology, Royal Hallamshire Hospital, Sheffield Teaching Hospitals, Sheffield, UK; 2 Department of Gastroenterology, Bradford Royal Infirmary, Bradford, UK; 3 Department of Gastroenterology, Whipps Cross University Hospital, London, UK; 4 Department of Gastroenterology, Addenbrooke’s Hospital, Cambridge, UK; 5 Department of Gastroenterology, Hull Royal Infirmary, Hull, UK; 6 Academic Unit of Pathology, Department of Neuroscience, Faculty of Medicine, Dentistry & Health, The University of Sheffield, Sheffield, UK; 7 Department of Gastroenterology, Northern General Hospital, Sheffield Teaching Hospitals, Sheffield, UK

**Keywords:** Coeliac disease, Small intestine, Endoscopy, Histopathology, Iron deficiency anaemia, Diagnostic tests, Health economics, Screening

## Abstract

**Background:**

International guidelines recommend coeliac serology in iron deficiency anaemia, and duodenal biopsy for those tested positive to detect coeliac disease. However, pre-endoscopy serology is often unavailable, thus committing endoscopists to take routine duodenal biopsies. Some endoscopists consider duodenal biopsy mandatory in anaemia to exclude other pathologies. We hypothesise that using a point of care test at endoscopy could fill this gap, by providing rapid results to target anaemic patients who require biopsies, and save costs by biopsy avoidance. We therefore assessed three key aspects to this hypothesis: 1) the availability of pre-endoscopy serology in anaemia; 2) the sensitivities and cost effectiveness of pre-endoscopy coeliac screening with Simtomax in anaemia; 3) whether other anaemia-related pathologies could be missed by this targeted-biopsy approach.

**Methods:**

Group 1: pre-endoscopy serology availability was retrospectively analysed in a multicentre cohort of 934 anaemic patients at 4 UK hospitals. Group 2: the sensitivities of Simtomax, endomysial and tissue-transglutaminase antibodies were compared in 133 prospectively recruited patients with iron deficiency anaemia attending for a gastroscopy. The sensitivities were measured against duodenal histology as the reference standard in all patients. The cost effectiveness of Simtomax was calculated based on the number of biopsies that could have been avoided compared to an all-biopsy approach. Group 3: the duodenal histology of 153 patients presenting to a separate iron deficiency anaemia clinic were retrospectively reviewed.

**Results:**

In group 1, serology was available in 361 (33.8 %) patients. In group 2, the sensitivity and negative predictive value (NPV) were 100 % and 100 % for Simtomax, 96.2 % and 98.9 % for IgA-TTG, and 84.6 % and 96.4 % for EMA respectively. In group 3, the duodenal histology found no causes for anaemia other than coeliac disease.

**Conclusion:**

Simtomax had excellent diagnostic accuracy in iron deficiency anaemia and was comparable to conventional serology. Duodenal biopsy did not identify any causes other than coeliac disease for iron deficiency anaemia, suggesting that biopsy avoidance in Simtomax negative anaemic patients is unlikely to miss other anaemia-related pathologies. Due to its 100 % NPV, Simtomax could reduce unnecessary biopsies by 66 % if only those with a positive Simtomax were biopsied, potentially saving £3690/100 gastroscopies.

**Trial registration:**

The group 2 study was retrospectively registered with clinicaltrials.gov. Trial registration date: 13^th^ July 2016; Trial registration number: NCT02834429.

## Background

The prevalence of coeliac disease is approximately 1 % [1–5]. However, 75 % of cases remain undiagnosed [6], possibly due to its insidious onset, and patients do not always have symptoms. Moreover, the sensitivities of the endoscopic features of coeliac disease are limited as they may not always be present or easily recognised [7, 8]. One of the common presenting symptoms is anaemia, affecting 15–26.8 % of untreated patients [9, 10]. It usually results from malabsorption, leading to iron, folate, and B12 deficiency [11]. One way to increase the detection of coeliac disease is by screening individuals with iron deficiency anaemia, which affects 2–5 % of the general population in the developed world [12, 13]. At the endoscopy setting, 2.6–8.7 % of patients presenting with anaemia are diagnosed with coeliac disease, although the data is sparse and mainly from small cohorts [10, 14–18]. The current British Society of Gastroenterology (BSG) iron deficiency anaemia guidelines recommend routine screening for coeliac disease with tissue transglutaminase (TTG) and/or endomysial antibodies (EMA). This is based on the excellent negative predictive value of modern serological tests for coeliac disease. Individuals who are tested positive should then undergo a gastroscopy for duodenal biopsy to confirm the presence of coeliac disease [19]. Anecdotally, the availability and utilisation of coeliac serology prior to endoscopy appears to be highly variable, thus committing clinicians to take duodenal biopsies if serology results are unavailable. However, this is an expensive way of case detection. A recent Swedish study [10] showed that a routine duodenal biopsy strategy was ineffective, with a number needed to biopsy of 577 to detect one case of coeliac disease, spending more than €30,000 per case. In an attempt to target patients who require a duodenal biopsy, Hopper et al. [20] devised a clinical decision tool using a combination of pre-endoscopy serological testing and symptom assessment. This algorithm had a 100 % sensitivity and negative predictive value in detecting coeliac disease when applied to 2000 prospectively recruited patients. Yet, the lack of serology availability prior to endoscopy in real clinical practice seemed to have precluded the widespread utilisation of this effective and cost saving clinical decision tool.

One method of filling the gap of unavailable serology is by using a point of care test at the point of endoscopy. Several point of care tests are now commercially available for clinicians and patients to purchase, mostly detecting TTG antibodies. Simtomax, a new point of care  test for coeliac disease, is a finger prick test that provides rapid results within ten minutes. Simtomax detects coeliac disease with a unique combination of immunoglobulin A (IgA) and immunoglobulin G (IgG) antibodies against deamidated gliadin peptides (DGP) as well as the total level of IgA [21]. This ensures that results are not affected by patients with IgA deficiency, which is more common in people with coeliac disease than the general population (2.6 % versus 0.14–0.2 %) [22].

In this study, our aim was to evaluate the role of utilising a pre-endoscopy point of care test for coeliac disease, Simtomax, in iron deficiency anaemia in a cost saving model. Firstly, we reviewed the rates of adherence to the BSG guidelines on coeliac serological screening in iron deficiency anaemia in real clinical practice, to demonstrate the pre-endoscopy availability of serology. We then ascertained the sensitivities of Simtomax in detecting coeliac disease in iron deficiency, and established the economic impact of using Simtomax as a screening tool to target biopsy taking only in those tested positive for Simtomax. Finally, we explored whether routine duodenal biopsy would yield any alternative causes for iron deficiency anaemia other than coeliac disease, in order to evaluate whether using Simtomax to target biopsies only in Simtomax positive anaemic patients would miss other duodenal pathologies causing anaemia. We chose to review the duodenal histology of patients from the general population attending a non-coeliac specialised iron deficiency anaemia clinic at the Northern General Hospital, because the results would represent real world data without tertiary referral bias.

## Methods

### Study design and participants

Group 1 was a multicentre retrospective analysis of all patients with anaemia attending a gastroscopy with duodenal biopsy at four UK hospitals (Addenbrooke’s, Bradford, Hull and Whipps Cross) over a 12 month period ranging from 2012 to 2014. The availability of coeliac serology prior to gastroscopy was reviewed.

Group 2 was a prospective study comparing the sensitivities of Simtomax to conventional serology in an iron deficient cohort. We prospectively recruited 133 consecutive patients (age range: 18–89 years, median 53) with iron deficiency with or without anaemia attending a single coeliac disease research endoscopy list at the tertiary referral centre Royal Hallamshire Hospital between 2013 and 2015. All recruited patients were consented for the study prior to the gastroscopy. The patients concurrently undertook the point of care test, Simtomax, conventional coeliac serology (IgA-TTG, IgA-EMA) and total IgA levels at the endoscopy unit. All patients then had a gastroscopy with quadrantic duodenal biopsy from the second part of the duodenum and at least one duodenal bulb biopsy. Patients were excluded from the study if they were known to have coeliac disease or were on a gluten free diet. Patients with coagulopathy, active gastrointestinal bleeding or a suspected carcinoma observed during the examination were also excluded. Clinical information of the patients was available to the endoscopist, however the endoscopist was blinded to the results of the Simtomax test.

Group 3 was a retrospective histological analysis of patients attending a separate non-coeliac specific iron deficiency anaemia clinic at the Northern General Hospital in 2013–2014. We reviewed their duodenal histology and hospital case notes to determine the yield of alternative causes other than coeliac disease in the context of iron deficiency anaemia.

### Point of care test, Simtomax

Simtomax is a point of care test for coeliac disease manufactured by Augurix Diagnostics, Switzerland. It detects both IgA and IgG antibodies to DGP, as well as the total IgA level. The assay is based on lateral flow immunochromatography using colloidal gold antihuman antibodies as a signal detector. A sample of 25 μl of capillary venous blood is required which can be obtained through a simple finger prick technique. The assay can also be performed using a plasma sample either in ethylenediaminetetraacetic acid (EDTA) or heparin as well as a separated serum sample, although a smaller sample volume of 20 μl is required. The blood sample is then applied to the test device, followed by the application of 5 drops of the provided buffer solution. The result can be read after 10 min. Positive results are indicated by the presence of a solid red test line for IgA and/or IgG-DGP positivity. A second single red line indicates the presence of IgA. An in-built red control line ensures a correctly functioning test.

### Serology

Total IgA was measured on a Behring BN2 nephelometer. IgA-TTG antibodies were evaluated using enzyme-linked immunosorbent assay kits (Aesku Diagnostics, Wendelsheim, Germany). An IgA-TTG titre of > 15 U/ml before 20/5/2014, a new cut off level of >9 U/ml from 20/5-11/12/2014, and then >7 U/ml from 12/12/2014 onwards, were regarded as positive as per the manufacturer’s guidance. IgA-EMA was detected by immunofluorescence on primate oesophagus sections (Binding Site, Birmingham, UK).

### Biopsies and histology

In total, at least five biopsies were taken from the duodenum, including at least one from the duodenal bulb, with each biopsy fixed in formalin at the time of the gastroscopy. Specimens were then processed, orientated and embedded in paraffin wax by the pathology department. Standard 3 μm thick sections at three levels were stained with haematoxylin and eosin, and reported by gastrointestinal histopathologists without knowledge of the Simtomax results. Villous atrophy was graded according to the modified Marsh criteria.

### Diagnosis of coeliac disease

The presence of villous atrophy (Marsh 3a-3c) on histology with a positive IgA-EMA or IgA-TTG were required for the diagnosis of coeliac disease. In cases of seronegative villous atrophy, human leucocyte antigen (HLA) genotyping was performed, with a negative HLA DQ2 or DQ8 phenotype used to rule out coeliac disease. Supporting information such as family history and response to a gluten free diet were also taken into account.

### Ethics

The study protocol was approved by the Yorkshire and the Humber Research Ethics committee and registered with the local research and development department of Sheffield Teaching Hospital NHS Foundation Trust under the registration number STH15416. Written consent was obtained from all participants.

### Statistical analysis

The sensitivity, specificity, positive (PPV) and negative predictive values (NPV) of Simtomax, IgA-EMA and IgA-TTG were measured against duodenal histology as the reference standard. As the PPV and NPV of a test are influenced by the pre-test probability, positive (PLR) and negative likelihood ratios (NLR) were also calculated as these are less prone to influence by disease prevalence. Exact Clopper-Pearson method was used to calculate the confidence intervals for the diagnostic test sensitivities.

### Cost analysis

In the UK, the Health Resource Group tariff (payment from the National Health Service [NHS] commissioners to hospitals for providing a service) for a gastroscopy with duodenal biopsy and a gastroscopy alone are £382 and £344 respectively. This means that £38 would be saved for the NHS budget if a duodenal biopsy was avoided for each gastroscopy performed. On the other hand, there is cross charging between departments for each service provided. At the Royal Hallamshire Hospital, the histopathology department charges the gastroenterology department £86 for the service of analysing four D2 biopsies and one D1 biopsy (local tariff may vary among different trusts). We will demonstrate the financial impact at a local and national level if Simtomax were to be used to target biopsies in iron deficient patients with a positive Simtomax test, as opposed to a routine biopsy strategy.

## Results

In group 1, a total of 934 patients with anaemia underwent a gastroscopy with duodenal biopsy at four UK hospitals. Coeliac serology was only available in 315 patients (33.8 %) prior to endoscopy. Forty-four (14 %) serology samples were performed in primary care.

**Fig. 1:**
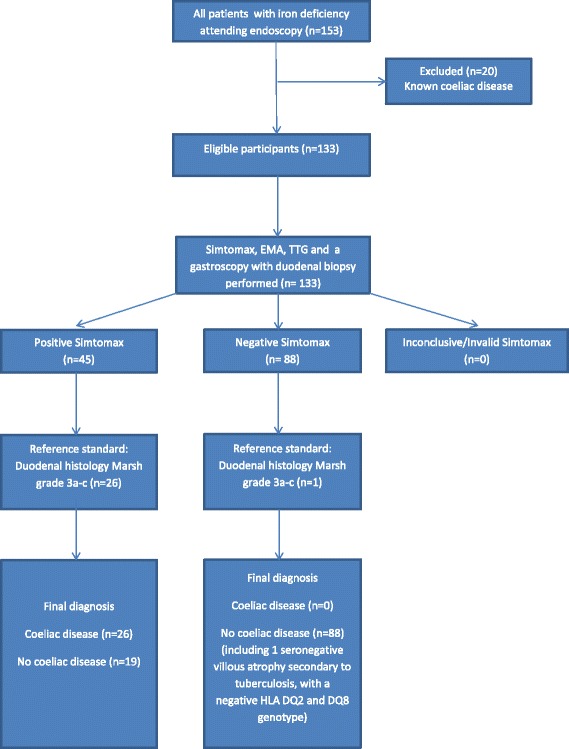
Flow of participants in group 2 study

In group 2, 133 patients (88 females) with iron deficiency attending for a gastroscopy at the tertiary referral centre Royal Hallamshire Hospital were prospectively recruited. Twenty-six patients (19.5 %) were diagnosed with coeliac disease. Simtomax correctly identified all cases of coeliac disease, defined by a combination of a positive serology (IgA-EMA/IgA-TTG) and Marsh grade 3a-c villous atrophy. The results are shown in Tables 1 and 2. There was one case of a 38 year old Zambian lady with seronegative villous atrophy secondary to tuberculosis. The sensitivity, specificity, PPV and NPV of Simtomax were 100 %, 82.2 %, 57.8 % and 100 % respectively. A comparison of the sensitivities of Simtomax, IgA-TTG and IgA-EMA are shown in Table 3. Please refer to Fig. 1 for the flow diagram of group 2. There were no invalid Simtomax results. No adverse events occurred with the Simtomax tests or gastroscopies.

**Table 1:** Group 2 patient characteristics and corresponding results

	Simtomax positive	EMA positive	TTG positive	M^a^0	M1	M2	M3a	M3b	M3c	Coeliac disease
Iron deficiency anaemia (*n* = 81)	30	13	19	55	7	1	2^b^	5	10	16 (19.8 %)
Iron deficiency without anaemia (*n* = 52)	15	10	15	33	8	1	2	2	6	10 (19.2 %)

^a^Marsh grade

^b^One patient had seronegative Marsh grade 3a villous atrophy which was secondary to tuberculosis instead of coeliac disease

**Table 2:** Cross tabulation of Simtomax results by serology and duodenal histology results

	Simtomax positive	Simtomax negative
Coeliac disease (positive serology and Marsh 3 histology)	26	0
No coeliac disease (negative serology and/or Marsh 0-2 histology)	19	88

**Table 3:** A comparison of the sensitivities of Simtomax, IgA-TTG and IgA-EMA in group 2

	Sensitivity, % (95 % CI)	Specificity, % (95 % CI)	PPV, % (95 % CI)	NPV, % (95 % CI)
Simtomax	100 (86.8–100.0)	82.2 (73.7–89.0)	57.8 (42.2–72.3)	100 (95.9–100.0)
IgA-TTG	96.2 (80.4–99.9)	91.5 (84.5–96.4)	73.5 (55.6–87.1)	99.0 (94.5–100.0)
IgA-EMA	84.6 (65.1–95.6)	99.1 (94.9–100.0)	95.7 (78.1–99.9)	96.4 (91.0–99.0)

**Fig. 2:**
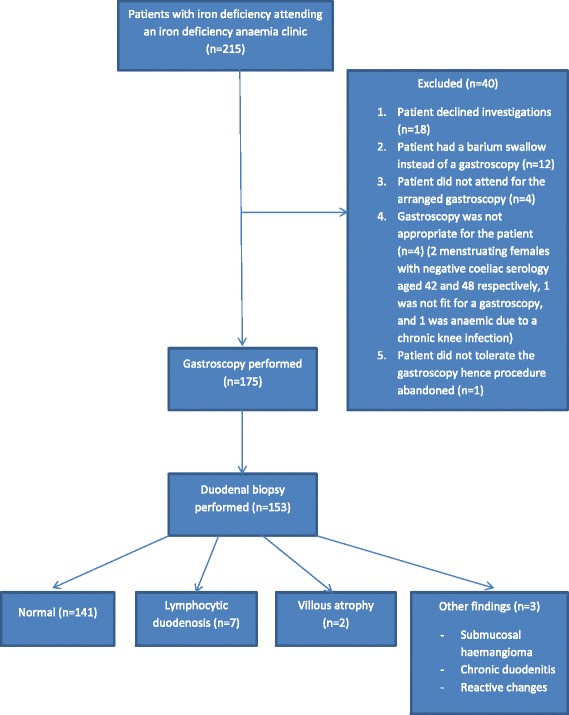
Flow chart for group 3 cohort analysis

In group 3, 215 patients with iron deficiency anaemia attended a separate non-coeliac specialised anaemia clinic at the Northern General Hospital for investigation from 2013–2014. A total of 175 patients underwent a gastroscopy, and 153 of those had a duodenal biopsy. The duodenal histology samples of these 153 patients were analysed. Two patients had Marsh grade 3 villous atrophy on histology- one had a positive coeliac serology and hence was diagnosed with coeliac disease; the other patient was found to have a colonic tumour during the course of the IDA investigation and subsequently died. He never had coeliac serology or HLA genotyping to confirm the diagnosis. Assuming the latter case to be coeliac disease, the prevalence of coeliac disease in group 3 would be 1.3 %. One hundred and forty-one patients (92.2 %) had normal duodenal histology. Seven patients (4.6 %) had lymphocytic duodenosis (Marsh grade 1) on their histology, all of whom had negative coeliac serology. We reviewed their hospital case notes and screened for drug causes for lymphocytic duodenosis such as aspirin, proton pump inhibitors, olmesartan, non steroidal anti-inflammatories and chemotherapy; autoimmune associations such as type 1 diabetes, thyroid disorders etc; and infections such as Helicobacter pylori, Whipple’s, Giardia etc. Six of these patients had a cause for or association with lymphocytic duodenosis: vitiligo, autoimmune hypothyroidism, multiple sclerosis, aspirin use, proton pump inhibitor use and Helicobacter pylori infection respectively. We could not find a cause attributable to the lymphocytic duodenosis in the remaining one patient, whose helicobacter status was unknown. Three patients had reactive changes, chronic duodenitis and submucosal haemangioma respectively on their duodenal histology which were not the cause for their iron deficiency anaemia. Please refer to Fig. 2 for the flow chart of group 3 patients.

### Cost saving economic model

In our group 2 cohort, 88 out of 133 patients had a negative Simtomax test. Based on the 100 % sensitivity and NPV of Simtomax, a duodenal biopsy could have been avoided in these 88 patients (66.2 %). At the Royal Hallamshire Hospital, the cost of having duodenal biopsies reported (four D2 and one D1 biopsy) is £86, and the price of each Simtomax test kit is £20. The cost saving from avoided biopsies in this cohort would be £7568 (£86x88). After taking into account the cost of using Simtomax on all patients (£20x133 = £2660), the overall cost saving in this cohort would be £4908 (£7568–£2660) for the gastroenterology department. This equates to a potential cost saving of £3690 per 100 gastroscopies. At a national level, the difference in the HRG tariff between a gastroscopy alone and a gastroscopy with duodenal biopsy paid by the clinical commissioners to the trust is £38. This equates to a cost saving of £2514/100 gastroscopies for the NHS budget (£38x88/133x100).

## Discussion

### Principal findings

We demonstrated with real life data that the availability of coeliac serology in anaemia prior to gastroscopy was low (33.8 %). This result is consistent with a study conducted by Wiland et al. in 2013 demonstrating that only one third of patients suspected to have coeliac disease had serology available prior to endoscopy [23].

To our knowledge, this is the first study showing that a combined IgA/IgG-DGP based point of care test, Simtomax, had 100 % sensitivity and negative predictive value in detecting coeliac disease in iron deficient patients. The positive and negative likelihood ratios were 5.63 and 0 respectively, indicating a negative Simtomax test effectively rules out coeliac disease in iron deficient patients. This compares to the results from the study by Mooney et al. which demonstrated a slightly lower but still excellent sensitivity of Simtomax at 92.7 % in an unselected cohort of 508 patients attending for a gastroscopy [24]. The higher sensitivity of Simtomax in this study could be due to the specific target population with iron deficiency anaemia causing a positive ascertainment bias. Although the pre-test probability in group 2 was relatively high, it is similar to that of Mooney et al.’s study at 13.4 %.

Our group 3 duodenal histology review revealed no alternative causes for iron deficiency anaemia other than villous atrophy secondary to coeliac disease. With a 100 % NPV, taking a duodenal biopsy in patients with a negative Simtomax test would be highly unlikely to yield any diagnosis for iron deficiency in routine clinical practice, and hence could be avoided in patients presenting with iron deficiency with or without anaemia.

The local cost saving of £3690/100 gastroscopies was based on the coeliac disease prevalence of 19.5 % in our coeliac enriched group 2 cohort. In a lower prevalence population, the potential cost saving would be greater. If we base our calculations on the average coeliac disease prevalence of 5 % in iron deficient cohorts, [14–18] the potential cost saving for the gastroenterology department would be £5826/100 gastroscopies, assuming the same tariff was applied; and £3456/100 gastroscopies would be saved at a national level for the NHS budget. This is excluding a wide range of intangible savings from the positive knock on effects, such as cost savings from not using biopsy pots and forceps, staff time and workload for both the endoscopy and histopathology departments.

### Strengths and weaknesses

The strength of our prospective study (group 2) is that the sensitivities of Simtomax were measured against duodenal histology rather than serology as the reference standard. Furthermore, in order to reduce selection bias, all patients underwent duodenal biopsies irrespective of their serology or Simtomax results. This sets our study apart from the three out of four published Simtomax studies, [25–27] where only patients with a positive serology or Simtomax test went on to have duodenal biopsies, which could potentially lead to a positive ascertainment bias and falsely elevated sensitivities. This limitation is also common in previous studies on the sensitivities of coeliac serology. A meta-analysis [28] published in 2010 on studies comparing IgA-DGP serology to IgA-TTG serology showed that only two [29, 30] out of eleven studies were unlikely to have ascertainment bias, where both of these studies also biopsied controls.

One of the limitations of our study is the relatively high pre-test probability in group 2 being investigated at our tertiary referral centre for coeliac disease, giving a coeliac disease prevalence of 19.5 %. This referral bias may falsely increase the positive predictive value of Simtomax, although the weight of its negative predictive value may be strengthened.

Another limitation is that even though the non-specialist group 3 cohort represented real life data from the general population where no other causes for anaemia apart from coeliac disease were found on duodenal histology, it is a relatively small cohort and may not be representative of other populations. For instance, infective causes may be seen in other cohorts, and their prevalence varies from different populations and geographical regions. Two Turkish studies reported a 2 % prevalence of Giardia found on duodenal biopsies in their cohorts with iron deficiency anaemia [31, 32]. On the other hand, a German study found a 0.2 % prevalence of Giardiasis on routine duodenal biopsy in 1000 unselected patients attending for a gastroscopy, [33] and a study from the U.S. had a 0.3 % yield of Giardiasis on routine duodenal biopsy in 300 patients presenting with abdominal pain [34]. Therefore, the cost saving economic model through biopsy avoidance in iron deficient patients with a negative Simtomax test may not be applicable to populations where parasitic infections are common, as infective diagnoses may be missed.

In our group 2 tertiary centre iron deficient cohort, there was one case of seronegative villous atrophy secondary to tuberculosis. This is a rare cause of seronegative villous atrophy. It must be emphasised that the group 2 cohort does not reflect what is normally seen in routine clinical practice, as the Royal Hallamshire Hospital is a tertiary referral centre. Apart from coeliac disease and parasitic infections, rarer malabsorptive enteropathies with villous atrophy have been reported in other studies, such as in patients with Whipple’s disease, [35] graft versus host disease, [36] common variable immunodeficiency, [37, 38] autoimmune enteropathy, [39], and olmesartan associated enteropathy [40]. The literature has shown that patients with gastrointestinal parasitic infections and other rare enteropathies described above typically present with significant symptoms such as diarrhoea, abdominal pain and malnutrition, rather than solely with iron deficency anaemia [34, 37–39]. Therefore, in iron deficient patients where there is a high index of suspicion for other enteropathies, such as malasborptive symptoms or high risk ethnicities, the threshold for taking duodenal biopsies should be lowered.

Our study demonstrated excellent sensitivity and negative predictive value of Simtomax in iron deficiency, and its performance was comparable to both IgA-EMA and IgA-TTG. This is consistent with two other studies testing Simtomax in high risk groups performed by Benkebil et al. in 2013 [26] (100 % sensitivity for coeliac disease in a high risk population) and Bienvenu et al. in 2014 [27] (100 % sensitivity and NPV for coeliac disease in IgA deficient children, median age 8.4). However, Bienvenu et al. 2012 [25] showed that the sensitivity of Simtomax to be slightly lower at 93.1 % when tested on a paediatric population with clinical suspicion of coeliac disease, although it should be noted that this sensitivity was measured against TTG as the reference standard rather than duodenal histology.

## Conclusion

This is the first study that demonstrated the excellent diagnostic accuracy of Simtomax in iron deficiency, which was comparable to conventional serological testing. With a 100 % sensitivity and negative predictive value, Simtomax could be used judiciously by clinicians as an effective and cost saving screening test for coeliac disease in the endoscopic setting, by avoiding duodenal biopsies in patients presenting with iron deficiency with a negative Simtomax test.

## Abbreviations

BSG: British society of gastroenterology
DGP: Deamidated gliadin peptide antibodies
EDTA: Ethylenediaminetetraacetic acid
EMA: Endomysial antibodies
HLA: Human leucocyte antigen
IgA: Immunoglobulin A
IgG: Immunoglobulin G
NHS: National Health Service
NLR: Negative likelihood ratio
NPV: Negative predictive value
PLR: Positive likelihood ratio
PPV: Positive predictive value
TTG: Tissue transglutaminase antibodies
