# Erratum to: ‘Pre-endoscopy point of care test (Simtomax- IgA/IgG-Deamidated Gliadin Peptide) for coeliac disease in iron deficiency anaemia: diagnostic accuracy and a cost saving economic model’

**DOI:** 10.1186/s12876-016-0536-y

**Published:** 2016-10-05

**Authors:** Michelle Shui Yee Lau, Peter D. Mooney, William L. White, Victoria Appleby, Sulleman Moreea, Ismail Haythem, Joshua E. Elias, Kiran Bundhoo, Gareth D. Corbett, Liam Wong, Her Hsin Tsai, Simon S. Cross, John M. Hebden, Sami Hoque, David S. Sanders

**Affiliations:** 1 Academic Department of Gastroenterology, Royal Hallamshire Hospital, Sheffield Teaching Hospitals, Sheffield, UK; 2 Department of Gastroenterology, Bradford Royal Infirmary, Bradford, UK; 3 Department of Gastroenterology, Whipps Cross University Hospital, London, UK; 4 Department of Gastroenterology, Addenbrooke’s Hospital, Cambridge, UK; 5 Department of Gastroenterology, Hull Royal Infirmary, Hull, UK; 6 Academic Unit of Pathology, Department of Neuroscience, Faculty of Medicine, Dentistry & Health, The University of Sheffield, Sheffield, UK; 7 Department of Gastroenterology, Northern General Hospital, Sheffield Teaching Hospitals, Sheffield, UK

## Erratum

Unfortunately, after publication of this article [1] it was noticed that several authors were missing their middle initials. The corrected author list can be seen above and the original article has been updated to reflect this change.
